# Pre-alerts from critical care ambulances to trauma centers: a quantitative survey of trauma team leaders in Ontario, Canada

**DOI:** 10.1186/s13049-024-01296-w

**Published:** 2024-12-19

**Authors:** Tara Williams, Brodie Nolan, Melissa McGowan, Tania Johnston, Sonja Maria, Johannes von Vopelius-Feldt

**Affiliations:** 1 Ornge, Mississauga, ON Canada; 2 Division of Emergency Medicine, Temerty Faculty of Medicine, University of Toronto, Toronto, ON Canada; 3 Department of Emergency Medicine, St. Michael’s Hospital Toronto, Unity Health Toronto, Toronto, ON Canada; 4 Li Ka Shing Knowledge Institute, St. Michael’s Hospital, Unity Health Toronto, Toronto, ON Canada; 5 School of Biomedical Sciences, Charles Sturt University, Bathurst, NSW Australia

**Keywords:** Pre-alert, Handover tool, Trauma, Aeromedical transport, Prehospital, Paramedic, Emergency, Communication, Patient handover, HEMS, Ornge

## Abstract

**Introduction:**

Pre-alerts from paramedics to trauma centers are important for ensuring the highest quality of trauma care. Despite this, there is a paucity of data to support best practices in trauma pre-alert notifications. Within the trauma system of Ontario, Canada, the provincial critical care transport organization, Ornge, provides pre-alerts to major trauma centers, but standardization is currently lacking. This study examined the satisfaction of trauma team leaders’ (TTLs) satisfaction with current trauma pre-alerts and their preferences for logistics, content, and structure.

**Methods:**

This was a quantitative survey of TTLs at adult and pediatric trauma centers across Ontario, Canada. Recruitment was through email to trauma directors, with follow-up efforts to target low-response sites to achieve good geographical representation. The survey was completed online and contained a combination of single or multiple-choice questions, Likert scales and free text options.

**Results:**

In total, 79 TTLs from adult and pediatric lead trauma centers across Ontario responded to the survey, which took place over a 120-day period. The survey achieved good geographical representation. Given the current processes, TTLs describe moderate satisfaction with room for improvement (median score 3, IQR 3–4 on a 5-point Likert scale). Their overall preference was for timely and direct communication, with some concerns about multiple channels of communication around logistics. Most TTLs agreed on the important and less important content details found in common standardized framework tools. For structure, 28/79 TTLs strongly preferred the cognitive aid ATMIST, 13/79 preferred IMIST-AMBO, and 8/79 preferred MIST or SBAR as the most useful.

**Conclusions:**

There is room for improvement through standardizing communication and streamlined pre-alert channels. Some disagreements exist between TTLs, particularly regarding logistics. Further research should examine TTL satisfaction after implementing the change in the pre-alert notification framework, which can address localized issues through stakeholder meetings with individual TTLs.

**Supplementary Information:**

The online version contains supplementary material available at 10.1186/s13049-024-01296-w.

## Background

Effective communication between paramedics and hospital staff contributes to the early identification of clinical concerns and timely interventions fundamental for the successful resuscitation of critically injured patients [1]. In advanced prehospital care systems, pre-alerts are integral for incoming patients requiring prompt, specialist trauma care. When paramedics pre-alert the receiving emergency department (ED) of the pending arrival of a high-acuity and time-critical patient, the trauma team can prepare necessary resources, such as blood products, the operating theatre or other critical care interventions [2]. Although pre-alert notifications are accepted as essential for quality patient care, there is a paucity of data supporting best practices and considerable variation in pre-alert content, structure, and logistics across Canada and internationally [3, 4]. Additionally, research has shown that pre-alert information is frequently incomplete or inaccurate, resulting in over- or undertriage of trauma patients in the ED [5]. Standardized frameworks are known to improve the accuracy and efficiency of pre-alerts, with several different tools described in the literature [6]. Examples include the situation, background, assessment, and recommendation (SBAR) and IMIST-AMBO acronyms. IMIST-AMBO denotes the identification of the patient, mechanism or medical complaint, injuries or information, signs and symptoms, treatment and trends, allergies, medications, background history, and other social information [4, 7]. Common pre-alert cognitive aids that have been described in the literature are outlined in Table 1.

**Table 1:**
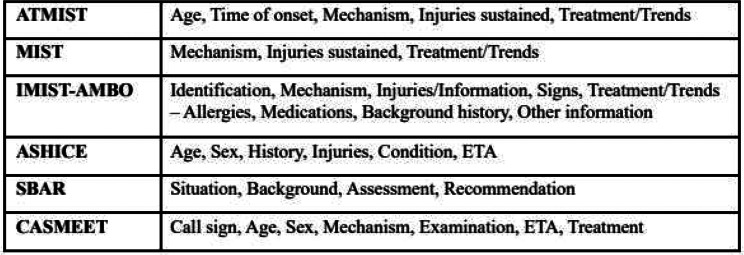
Common pre-alert cognitive aids

Ornge is the sole critical care transport provider in Ontario, Canada, and routinely transports severely injured patients to 13 regional trauma centers. At present, a standardized framework is not utilized within Ornge to report patient updates. Thus, paramedics currently provide pre-alerts to trauma centers that are typically ad hoc, inconsistent in format and conveyed using different communication routes, either directly to the ED or via dispatch. To improve the quality of pre-alerts and, ultimately, patient outcomes, this study aimed to understand Trauma Team Leaders’ (TTLs) preferences regarding the content, structure, and logistics of trauma pre-alerts. This information will guide the implementation of evidence-based changes in practice within the organization and add to the literature supporting trauma pre-alerts.

## Methods

This quantitative, descriptive study used an online cross-sectional survey to examine trauma pre-alert content, structure, and logistics preferred by TTLs at trauma centers across Ontario, Canada. A secondary aim was to assess TTL satisfaction with the current pre-alert process to provide a baseline for comparison following an anticipated change in practice informed by the findings of this research. In our observational study, we reported these findings following the STROBE guidelines [8].

### Study setting

**Fig. 1:**
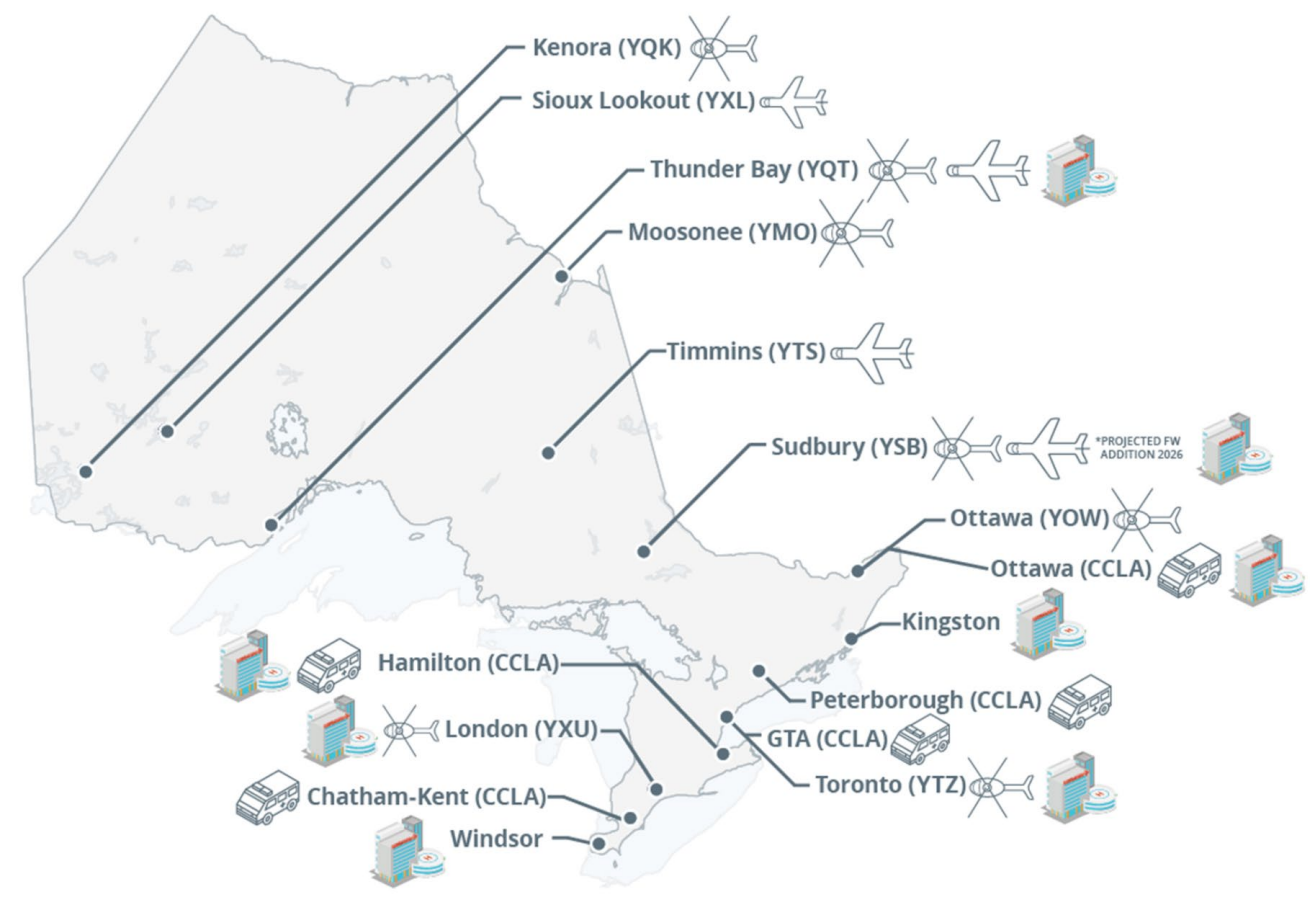
Map of Ornge critical care asset and trauma centre locations

This study was conducted in Ontario, the most populous province in Canada, with over 15 million inhabitants living in one million km^2^ [9]. The study location represents a significant portion of Canada’s trauma care system. Ornge is the province’s sole provider of critical care and air medical transport, operating a fleet of twelve rotor-wing and eight fixed-wing aircraft. Each resource is staffed with two advanced care or critical care paramedics. Assets are allocated based on the appropriate level of care required and the nearest base with the quickest resources [10]. The population density in Ontario is significantly greater in the southern part of the province. In contrast, many communities within the vast geography of Northern Ontario have very low density and are remote. Thus, timely access to definitive hospital care and trauma centers is challenging. Ontario has 13 adult and pediatric regional trauma centers that receive patients from Ornge (Fig. 1).

Paramedics respond to the scene of injury, stabilize, and transport injured patients directly to a trauma center. Alternatively, patients may be conveyed to a local hospital by land emergency medical services (EMS) for stabilization, and Ornge may perform a secondary transfer or ‘modified scene call.’ Table 2 provides an overview of trauma centers and patient transports by Ornge in 2022–2023. Regardless of the mission type, paramedics communicate en route via two-way radio or satellite phone to provide a pre-alert to the receiving ED. Currently, paramedics contact the on-call Ornge Transport Medicine Physician (TMP), who relays the pre-alert information to the TTL at the receiving hospital. In parallel, communications officers in the Ornge Control Centre (OCC) also relay details to the ED charge nurse. No formal structure or framework guides the pre-alert process, and there is no direct communication between the Ornge paramedics and the receiving trauma centre.

**Table 2:**
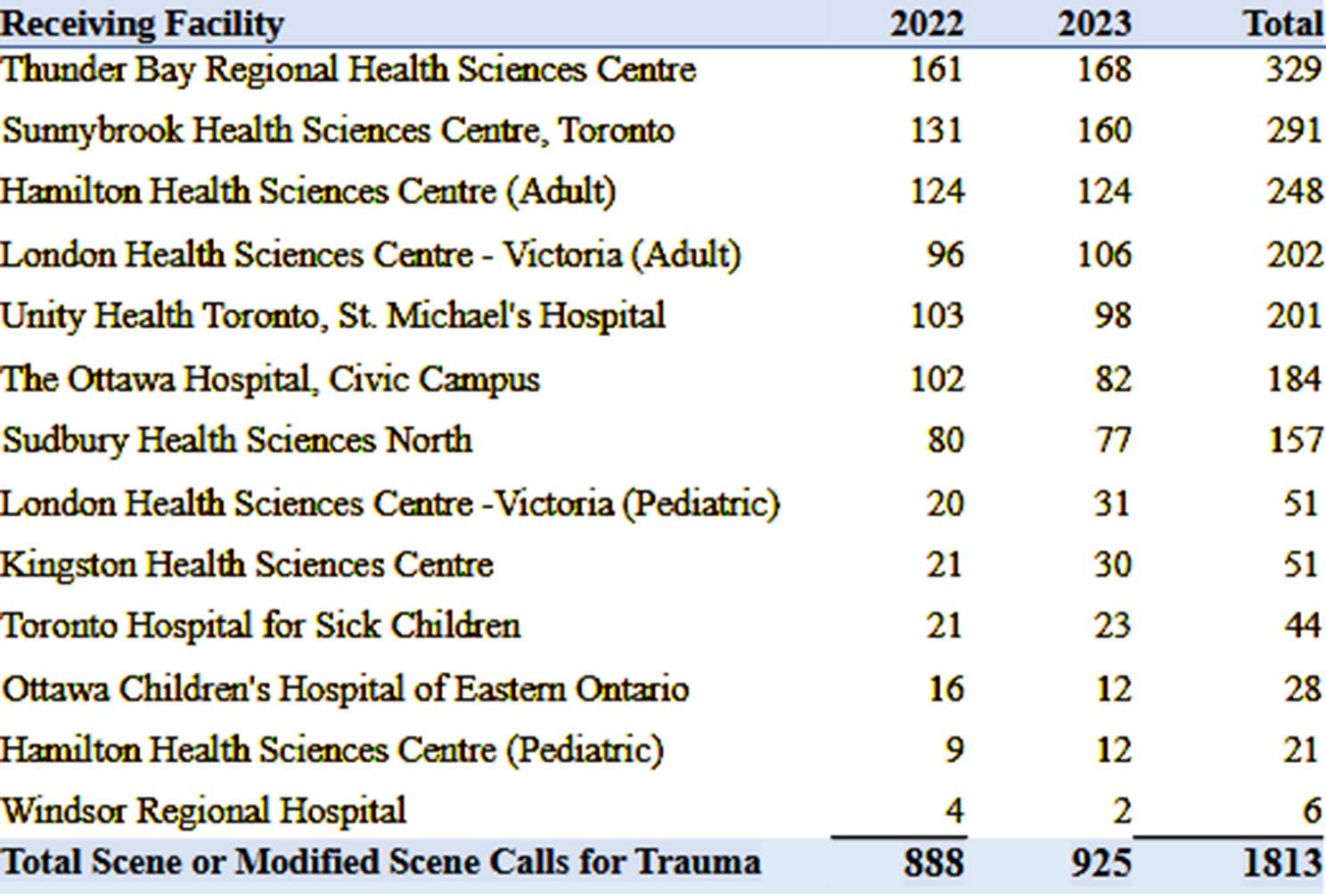
List of receiving trauma centres in Ontario and number of scene or modified scene calls for trauma

### Survey design

Following a focused literature review, we designed a purpose-built survey to address the following aspects: participant demographics, TTL preferences regarding trauma pre-alert logistics, structure, and content, and TTL satisfaction with current processes. We used a combination of single or multiple-choice questions, Likert scales, and free text. The initial survey was drafted by TW and JV with advice from BN. We then tested the survey with two emergency department physicians undergoing a fellowship in pre-hospital retrieval medicine with Ornge. Their feedback was used to inform survey modifications. The final survey consisted of 14 items (Appendix 1). Participants completed the survey online on a dedicated survey platform (Jotform^®^, San Francisco, CA, USA). The data were downloaded for analysis to a secure server at the research team’s organization. No directly identifiable information was collected from the participants.

### Participants and recruitment

The population for the study included TTLs across all 13 adult and pediatric trauma centers in Ontario, Canada. While there is no established record of the number of TTLs in the system, we estimated this number to be 250. We used a stratified random sampling technique for participants. Following interim reviews of response rates, we targeted follow-up efforts at sites with lower response rates. This method ensured that every TTL within the population had an equal chance of participating, thus reducing selection bias. Invitations to participate in the survey were emailed to all trauma directors in Ontario’s trauma centers, alongside participant information. The email requested that the trauma directors forward the documents to all TTLs at their respective sites. Reminder emails were sent at two-week intervals during the 120-day data collection period to improve response rates until we reached a pre-determined minimum sample size of 50 responses. Consent for participation was assumed through the completion of the survey.

### Data analysis

We used simple descriptive statistics to present the results of this quantitative survey. Categorical and binary results are presented as percentages and absolute numbers. Likert scales are presented as medians and interquartile ranges. If deemed relevant, free text was included in the results after alteration or censoring to ensure anonymity.

### Ethics approval

This study received ethics approval from the Unity Health Toronto, St. Michael’s Hospital Research and Ethics Board (study reference number 23 232) and the Charles Sturt University Human Research Ethics Committee.

## Results

We received 79 survey responses during the period from January to April 2024. Table 3 provides an overview of the demographics, and geographical distribution of the respondents.

**Table 3:**
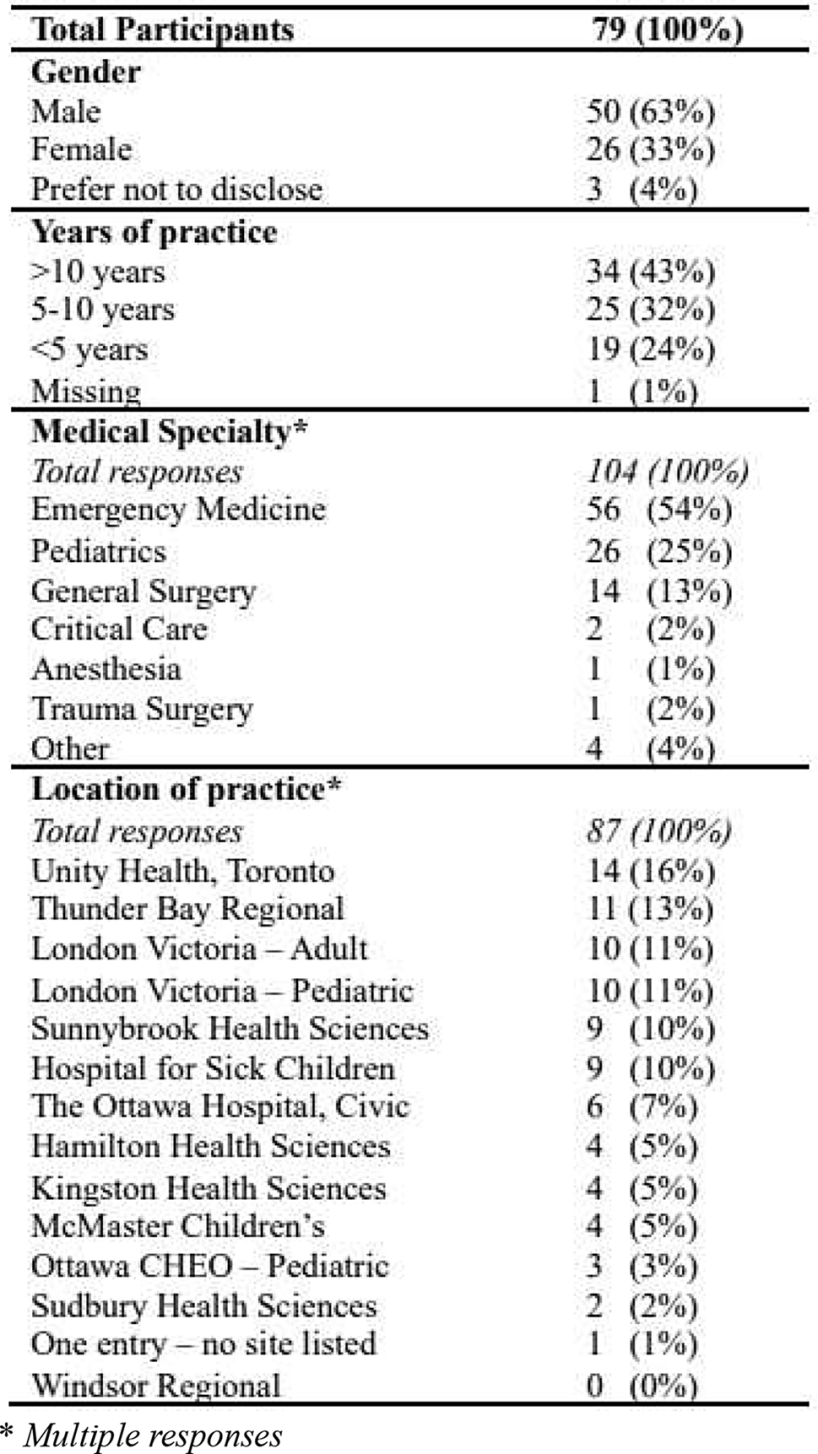
Demographics

**Fig. 2:**
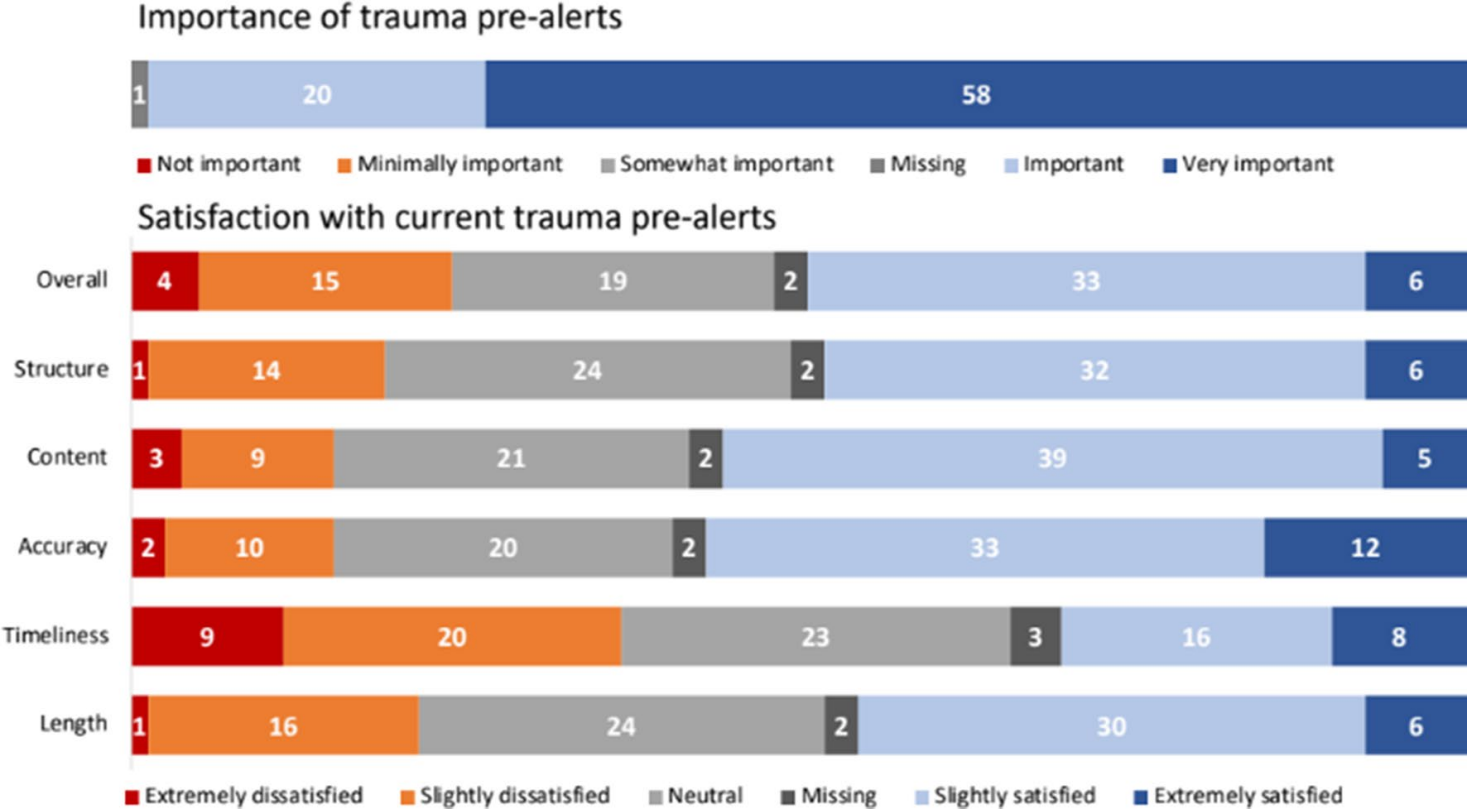
Trauma team leaders’ perceptions of pre-alert

**Fig. 3:**
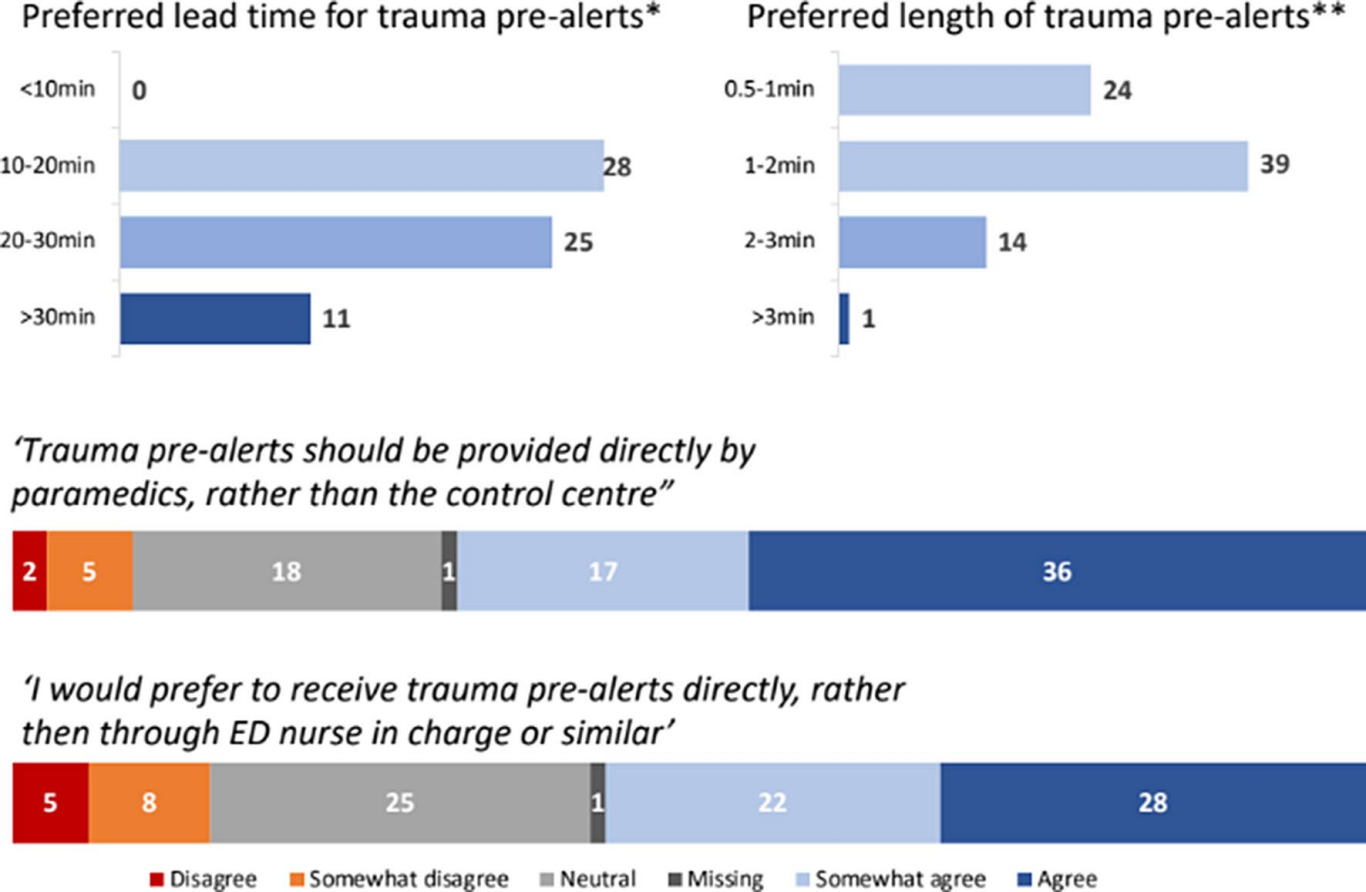
Trauma team leaders’ preferences for pre-alert logistics. *5 missing responses **1 missing response

Overall, 73% (58/79) of the TTLs categorized pre-alerts as very important and described moderate satisfaction with the current Ornge process with room for improvement (median score 3, interquartile range 3–4 on a 5-point Likert scale). Figure 2 outlines participant perceptions of pre-alerts, while Fig. 3 provides an overview of TTLs’ preferences for pre-alert logistics. Their overall preference was for timely and direct communication, with 35% (44/79) wanting pre-alerts 10–20 min before patient arrival, followed by 32% (25/79) of TTLs favouring 20–30 min. 49% (39/79) of TTLs preferred the length of the pre-alert to be 1–2 min. Concerns exist regarding multiple channels of communication and how the pre-alerts should occur. Participant free text responses included some TTLs wanting to speak to paramedics directly via radio to optimize trauma team preparedness. Others supported a consistent approach for pre-alerts to mitigate the redundancy of pre-alerts passed on from the ED nurse in charge, Ornge TMP, and OCC.

**Fig. 4:**
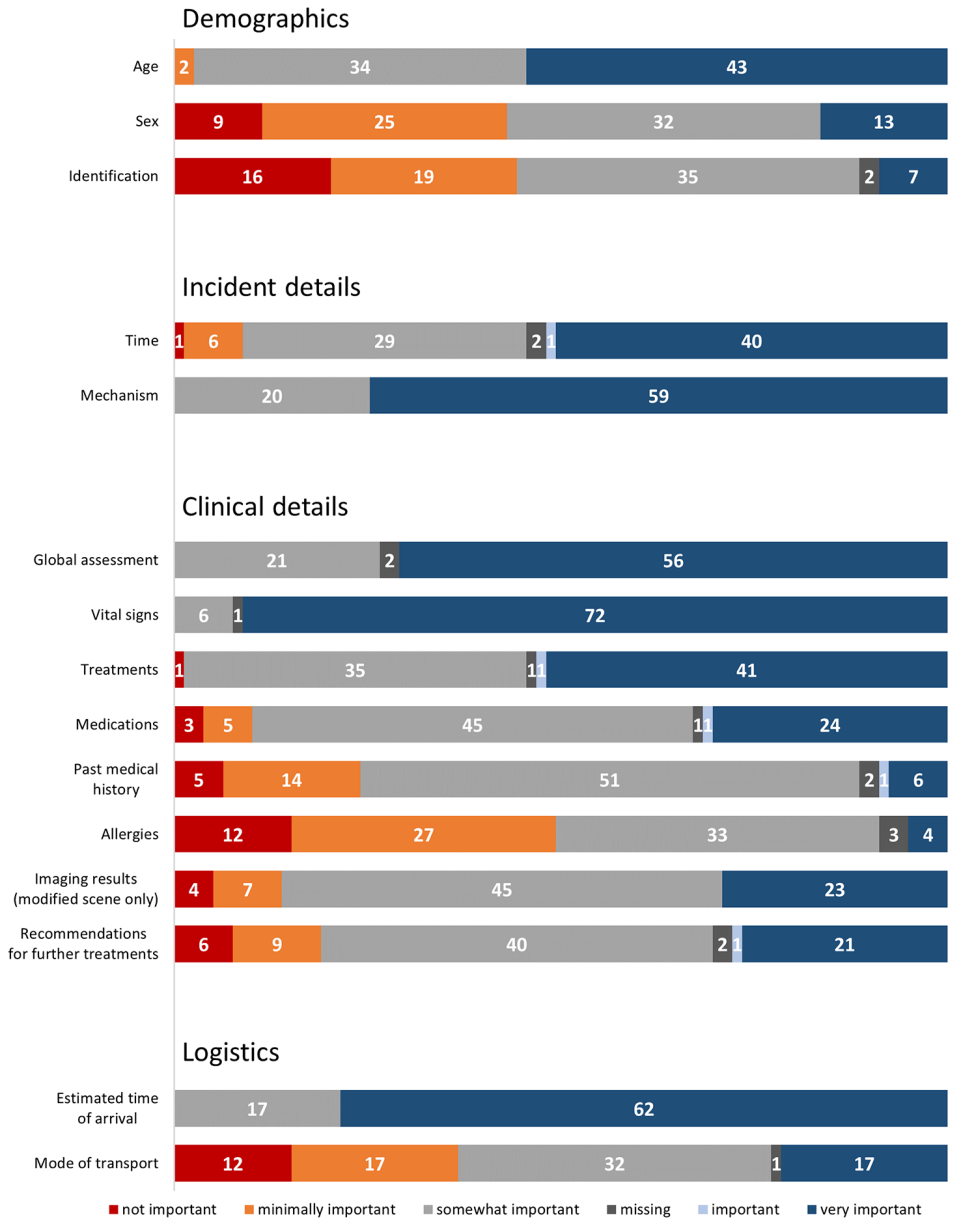
Trauma team leaders’ preferences for pre-alert content

The patient’s estimated arrival time at the trauma center was identified as very important information for 79% (63/79) of the TTLs. The content participants preferred to be included in a pre-alert is summarized in Fig. 4. Age was revealed to be the most useful demographic factor (54%, 43/79), with the mechanism of injury (75%, 60/79) and time of injury (51%, 40/79) as the most useful information to provide regarding the incident details. The most common clinical details included vital signs (91%, 72/79) and a global subjective assessment of patient stability ranging from hemodynamically stable to peri-arrest (71%, 56/79).

**Fig. 5:**
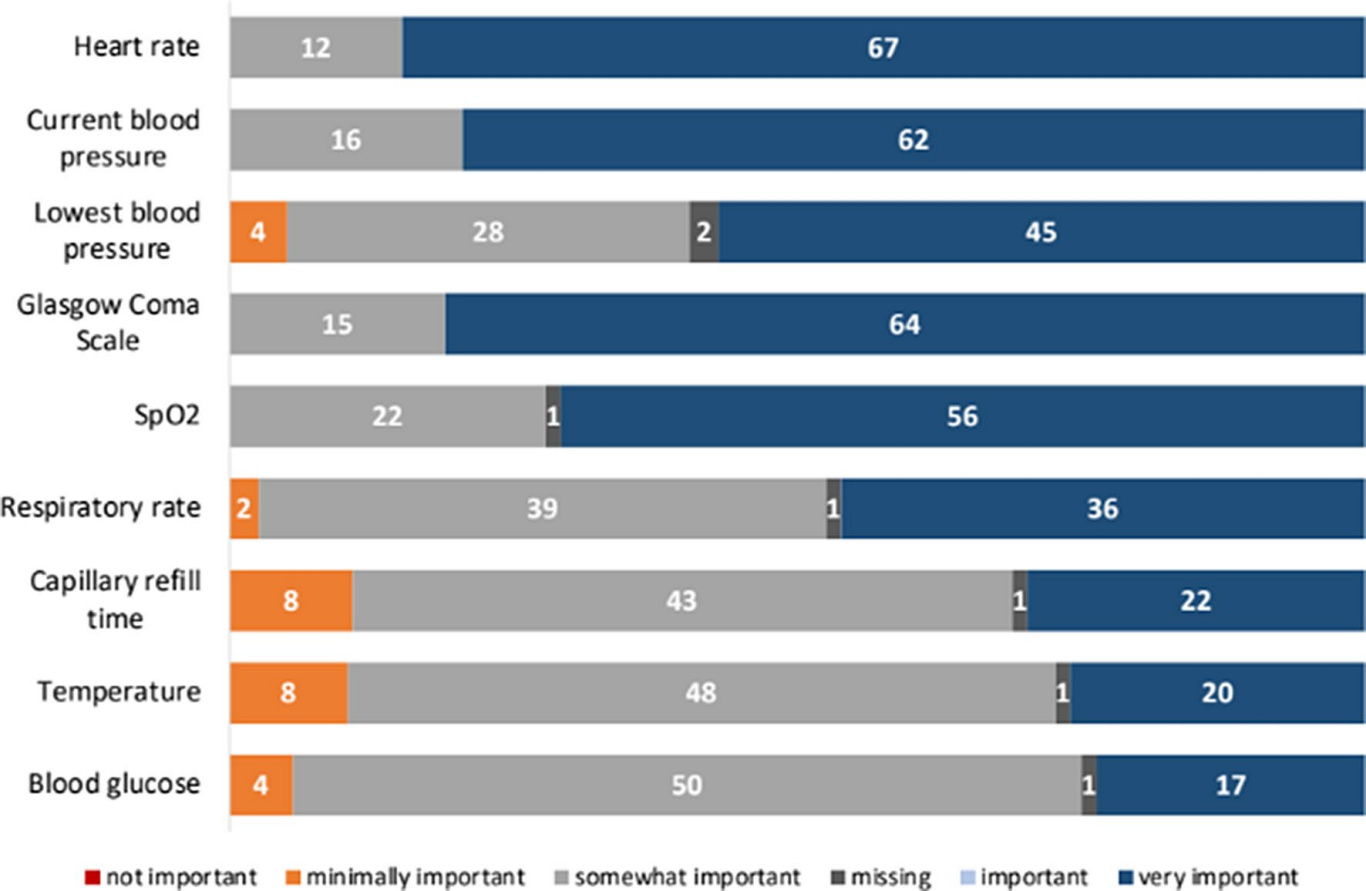
Trauma team leaders’ preference for vital signs included in pre-alert

As shown in Fig. 5, the vital signs identified as being the most important by TTLs included heart rate (84%, 66/79), followed by Glasgow Coma Scale (80%, 63/79) and current blood pressure (77%, 61/79).

**Fig. 6:**
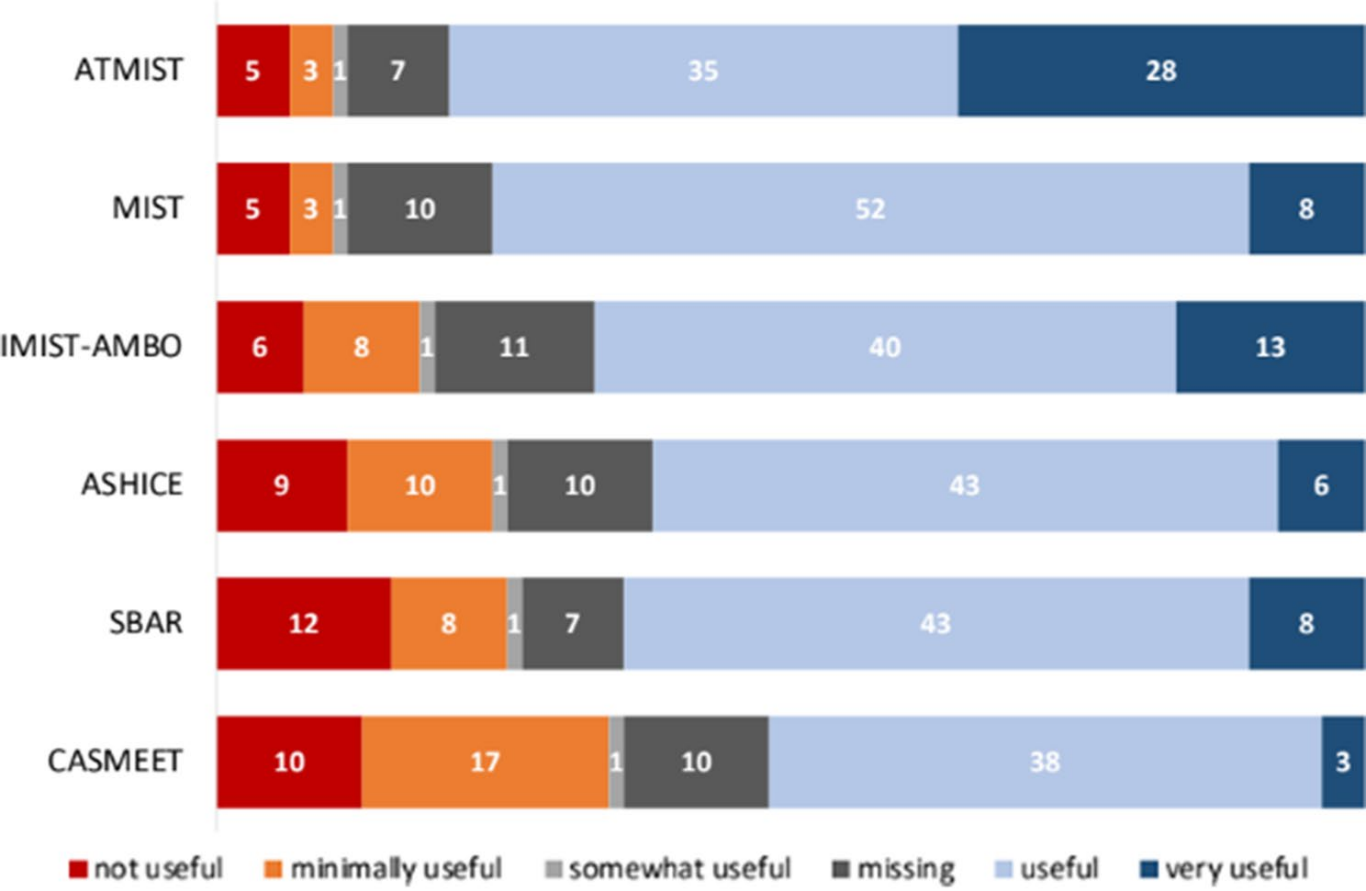
Preferences for cognitive aids

Most TTLs agreed on the important and less important content details found in common standardized framework tools. For structure, 35% (28/79) of TTLs expressed a strong preference for the cognitive aid ATMIST, followed by 17% (13/79) who preferred IMIST-AMBO, and 10% (8/79) who stated that MIST or SBAR was the most important (Fig. 6).

## Discussion

This descriptive, quantitative survey analysis of TTLs in all adult and pediatric lead trauma hospitals in Ontario is the first survey of this kind in North America. Good geographical representation and response rates were achieved throughout the province, especially between trauma centers in the higher population density areas of southern Ontario and those covering the more remote northern regions. In addition, response rates reflected a good balance of adult and pediatric voices. The key findings include moderate TTL satisfaction with the current trauma pre-alerts from Ornge, suggesting room for improvement. Regarding trauma pre-alert content, structure, and logistics, the survey provides useful insights into TTLs’ preferences, which can guide quality improvement interventions within Ornge and other EMS organizations. While specific research on trauma pre-alerts is limited, evidence exists on the use of structured frameworks during face-to-face handover from paramedics to hospital staff in the ED [11]. In this context, standardized framework tools are advantageous as cognitive aids for paramedics to consistently provide pertinent information despite the pressures of working in high-cognitive- load and resource-limited environments [12]. Barriers known to confound handovers in the ED include a lack of standardized processes, noisy and dynamic environments, organizational culture, and medical hierarchical structures [3, 13]. In the same way clinicians perform standard patient assessments, following a standardized handover mnemonic contributes to patient safety. Javidan et al. [7] reported that handovers performed without a framework tool use repetition, leading to a lack of active listening. As trauma pre-alerts share many of the challenges and opportunities of in-hospital handover, implementing similar standardized mnemonics would likely improve patient safety and system efficiency.

One mnemonic that paramedics use to facilitate handover in the ED is ATMIST, which is almost ubiquitous as both a pre-alert and in-hospital handover tool in the United Kingdom [14]. It contains the information ranked most consistently as important or very important by TTLs in our survey and, as such, would be a logical choice to trial for trauma pre-alerts in our organization. However, the implementation of new procedures needs to take into consideration pre-existing opportunities and limitations. In our study, the IMIST-AMBO was the second preferred tool for pre-alerts, after the ATMIST. Iedema et al. [15] found that IMIST-AMBO provided a logical sequence for information sharing and diminished repetition, thereby improving comprehension by the trauma team.

Moreover, a study of IMIST-AMBO conducted at Canada’s largest level-one trauma center demonstrated that the priority of information delivery aligns with the clinical criticality desired by ED staff [16]. This approach improved structure, flow, and duration while limiting the conveyance of unnecessary information [7]. The IMIST-AMBO framework has also been validated in an Australian trauma setting with a similar healthcare model and patient population density [17]. Before and during our study period, one adult trauma center and one pediatric trauma center adopted the IMIST-AMBO tool for the ED handover of trauma patients [16]. Therefore, to reduce variability and improve the probability of successful change management, working with these existing structures would be sensible. One such approach would be to use the IMIST part of the tool for pre-alert, while the full IMIST-AMBO is used for patient handover in the trauma bay. IMIST also largely overlaps with ATMIST, which was the highest-ranked tool in our survey. This process allows paramedics to complete pre-alerts and handovers of trauma patients with one tool, thus reducing cognitive load. This approach was previously described and favoured by paramedics and trauma team clinicians in a qualitative study by Evans et al. [18]. The outcomes from a study by Maddry et al. [19] validated this tool as conveying sufficient trauma pre-alert criteria for an appropriate duration. Two pieces of information not included in either ATMIST or IMIST-AMBO but were ranked highly by the TTLs in our survey are a global assessment of the severity of injuries (i.e. near dying, critical or stable) and the patient’s estimated arrival time. Both can be added to trauma pre-alert checklists, which can be provided to paramedics within a critical care transport organization.

Further suggestions for improvements from our survey relate to the logistical aspects of trauma pre-alerts. As in most time-critical and high-stakes scenarios, direct communication channels are usually preferable, which was confirmed in our survey. However, complexities arise with this practice as it requires TTLs to be paged through the trauma center switchboard. This process can take several minutes, which is impractical for paramedics providing care to critically injured patients. A possible solution to this issue would be to create a single number where TTLs can be contacted directly at each trauma center. This process will require extensive collaboration with individual trauma centers and serve as a reminder of change implementation, requiring stakeholder buy-in from all involved organizations [20]. Similarly, while this survey provides important information to guide change in practice from the perspective of TTLs receiving handovers, we will undertake stakeholder engagement and provide feedback opportunities from our paramedics before implementing any new process. Importantly, after change implementation and a settling-in period, we will repeat aspects of this survey to assess whether the implemented changes had the desired effects.

### Limitations

Limitations of our study include the exclusive focus on TTLs perceptions and preferences of trauma pre-alerts and the lack of a qualitative element. This focus on TTLs was not intended to diminish the importance of the multidisciplinary trauma team, in particular paramedics, ED nursing staff, dispatchers, and transport medicine physicians who provide logistical and medical support in the control centre. Due to logistical and research ethics limitations, we only had access to the TTL email distribution list and therefore focused on their perspective as the key receiver of information from trauma pre-alerts. While it is possible that other trauma team members would have had different preferences, it would not be practical to implement more than one handover structure to accommodate different trauma team members’ needs. As such, we believe that the focus on the TTL as the key information stakeholder is an appropriate approach to guide pre-alert content and structure in our system. Further research could use a mixed methods approach, adding qualitative interviews to ascertain a more profound understanding from participants of different backgrounds. For example, a focus group of paramedics, TTLs, RNs, and dispatchers could address the complexities involved in communication between mobile structures and heterogeneous providers in different regions of the province, while also allowing for interaction between the different stakeholders. While this approach would have certainly been interesting and provide meaningful results, it was outside the scope of this project. A final important limitation is the assumption that addressing TTLs’ trauma pre-alert preferences will actually result in improved processes. This assumption will be tested in future research, which will measure satisfaction as well as other key performance indicators such as accuracy and timeliness of information, following implementation of the standardized pre-alert structures outlined above.

## Conclusion

Pre-alerts are integral for safety and the continuity of care as various adverse outcomes can result from poor communication, influencing morbidity and mortality. There is a dearth of literature on pre-alerts in trauma surrounding best practices in HEMS transport. Consequently, there is room for improvement through standardizing communication and streamlining communication channels for trauma pre-alerts. Some disagreements exist between TTLs, particularly regarding logistics. Further research should examine TTL satisfaction after implementing the change in the pre-alert framework, which can address localized issues through stakeholder meetings with individual TTLs. Improved outcomes should prompt a more extensive analysis of trauma pre-alerts and include the effect of trauma patients on arrival at the hospital.

## Electronic supplementary material

Below is the link to the electronic supplementary material.

Supplementary Material 1

## Data Availability

Access to the research data would require both REB and a contract to share this.

## Abbreviations

TTLs: Trauma Team Leaders
ED: Emergency Department
SBAR: Situation, Background, Assessment, and Recommendation
IMIST-AMBO: Identification, Mechanism, Injuries, Signs, Treatment, Allergies, Medications, Background, Other information
TMP: Transport Medicine Physician
OCC: Ornge Control Centre
EMS: Emergency Medical Services
HEMS: Helicopter Emergency Medical Services
