# Supplementary material for: Pre-alerts from critical care ambulances to trauma centers: a quantitative survey of trauma team leaders in Ontario, Canada
Source: Scand J Trauma Resusc Emerg Med. 2024 Dec 19;32:134. doi: 10.1186/s13049-024-01296-w (PMC11660582; doi:10.1186/s13049-024-01296-w)
Supplement: Supplementary file 1 — Supplementary Material 1 [file 13049_2024_1296_MOESM1_ESM.docx]

**Appendix 1.** A needs assessment to determine the optimal pre-alert notification information for trauma patients

Trauma centers provide the highest level of care for severely injured patients and can mobilize multiple resources quickly. Timely, accurate and succinct information from prehospital providers to the receiving trauma centre is essential for ensuring that the response of the trauma center is efficient and effective. Pre-alert notifications for patients transported directly from a scene are a vital aspect of the prehospital-hospital trauma pathway interface because they allow sufficient time for trauma teams to assemble. As in many other high-stakes, variable and time-pressured situations, standardization of pre-alerts from paramedics to trauma centres has the potential to improve the quality of this process and to reduce the risk of patient harm from incomplete or incorrect information relay. While some trauma systems have standardized pre-alert contents and pathways, this is not true across trauma centers in Ontario, where Ornge transports trauma patients. This project aims to identify the preferred pre-alert content, structure, and pathway in the setting of Ornge critical care transports to trauma centers across Ontario.

1. **How important do you consider a pre-alert for trauma care?**

| **Not**  **Important**  **1** | **Minimally Important**  **2** | **Somewhat Important**  **3** | **Important**  **4** | **Very**  **Important**  **5** |
| --- | --- | --- | --- | --- |
|  |  |  |  |  |

Comments:

1. **How long prior to arrival would you ideally receive a pre-alert?**

- Less than 10 minutes out
- 10-20 minutes out
- 20-30 minutes out
- >30 minutes out

Comments:

1. **How long should the average pre-alert be?**

- 30 seconds – 1 minute
- 1 – 2 minutes
- 2 – 3 minutes
- > 3 minutes

Comments:

1. **I would prefer a direct pre-alert from paramedics rather than the dispatch/operations control centre.**

| **Disagree** | **Somewhat** **Disagree** | **Neutral** | **Somewhat** **Agree** | **Agree** |
| --- | --- | --- | --- | --- |
|  |  |  |  |  |

Comments:

1. **I would prefer a personal pre-alert to me (TTL) rather than the ED nurse in charge or similar.**

| **Disagree** | **Somewhat** **Disagree** | **Neutral** | **Somewhat** **Agree** | **Agree** |
| --- | --- | --- | --- | --- |
|  |  |  |  |  |

Comments:

1. **Please rate the importance of the following information to capture:**

|  | **Not Important** | **Minimally Important** | S**omewhat Important** | **Important** | **Very Important** |
| --- | --- | --- | --- | --- | --- |
| **Patient's Age** |  |  |  |  |  |
| **Gender** |  |  |  |  |  |
| **Time of injury** |  |  |  |  |  |
| **Mechanism of injury** |  |  |  |  |  |
| **Vital Signs** |  |  |  |  |  |
| **eFAST results (if applicable)** |  |  |  |  |  |
| **Chest X-ray results (if applicable)** |  |  |  |  |  |
| **Pelvic X-ray results (if applicable)** |  |  |  |  |  |
| **CT results (if applicable)** |  |  |  |  |  |
| **Treatment received** |  |  |  |  |  |
| **Global Assessment (peri-arrest to stable)** |  |  |  |  |  |
| **Past Medical History** |  |  |  |  |  |
| **Prescribed medications; on anticoagulants?** |  |  |  |  |  |
| **Allergies** |  |  |  |  |  |
| **Pt identifiers avail (name, date of birth)** |  |  |  |  |  |
| **Recommendations for specific interventions or specialties (i.e. anticipated need for transfusion, intubation or subspecialty consult)** |  |  |  |  |  |
| **Estimated time of arrival to you** |  |  |  |  |  |
| **Arrival by land or air** |  |  |  |  |  |

Comments:

**6a. For the list of primary injuries noted on patient assessment, would you prefer these to be structured according to:**

- - Head to toe
  - ABCDE
  - Severe to less severe
  - Other, please specify:

**6b. Which of the following vital signs are important to capture?**

|  | **Not Important** | **Minimally Important** | **Somewhat**  **Important** | **Important** | **Very Important** |
| --- | --- | --- | --- | --- | --- |
| **Heart Rate** |  |  |  |  |  |
| **Current BP** |  |  |  |  |  |
| **Lowest BP** |  |  |  |  |  |
| **GCS** |  |  |  |  |  |
| **Sp02** |  |  |  |  |  |
| **Resp Rate** |  |  |  |  |  |
| **Skin colour** |  |  |  |  |  |
| **Temperature** |  |  |  |  |  |
| **Blood Glucose** |  |  |  |  |  |

1. **Is there any other information you would like to see included in the pre-alert?**
2. **Please rate the following established pre-alert cognitive aids below:**

|  | **Not Important** | **Minimally Important** | **Somewhat Important** | **Important** | **Very Important** |
| --- | --- | --- | --- | --- | --- |
| **ATMIST** |  |  |  |  |  |
| **MIST** |  |  |  |  |  |
| **IMIST-AMBO** |  |  |  |  |  |
| **ASHICE** |  |  |  |  |  |
| **SBAR** |  |  |  |  |  |
| **CASMEET** |  |  |  |  |  |

a. ATMIST (Age, Time of onset, Mechanism, Injuries sustained, Treatment/Trends)

b. MIST (Mechanism, Injuries sustained, Treatment/Trends)

c. IMST-AMBO (Identification, Mechanism, Injuries or Information, Signs, Treatment/Trends - Allergies, Medications/Background history/Other information)

d. ASHICE (Age, Sex, History, Injuries, Condition, ETA)

e. SBAR (Situation, Background, Assessment, Recommendation)

f. CASMEET (Call sign, Age, Sex, Mechanism, Examination, ETA, Treatment)

1. **How satisfied are you with current trauma pre-alerts from Ornge?**

|  | **Extremely Dissatisfied** | **Slightly Dissatisfied** | **Neutral** | **Slightly**  **Satisfied** | **Extremely**  **Satisfied** |
| --- | --- | --- | --- | --- | --- |
| **Overall** |  |  |  |  |  |
| **Structure** |  |  |  |  |  |
| **Content** |  |  |  |  |  |
| **Accuracy** |  |  |  |  |  |
| **Timeliness** |  |  |  |  |  |
| **Length** |  |  |  |  |  |

Comments:

1. **Is there anything else you would like to share?**

**Please tell us a bit about yourself:**

1. **What is your medical background? Please choose all that apply.**

- Anaesthesia
- Emergency Medicine
- General surgery
- Pediatrics
- Other, please specify:

1. **What is your gender?**

- Male
- Female
- Other
- Prefer not to disclose

1. **How many years have you been in practice?**

- < 5 years
- 5-10 years
- > 10 years

1. **What hospital are you from?**

- Hamilton Health Sciences Centre (Adult)
- Hamilton Health Sciences Centre (Pediatric)
- Kingston Health Sciences Centre
- London Health Sciences Centre, Victoria Hospital (Adult)
- London Health Sciences Centre, Victoria Hospital (Pediatric)
- Ottawa Children's Hospital of Eastern Ontario
- Sudbury Health Sciences North
- Sunnybrook Health Sciences Centre, Toronto
- The Ottawa Hospital, Civic Campus
- Thunder Bay Regional Health Sciences Centre
- Toronto Hospital for Sick Children
- Unity Health Toronto, St. Michael's Hospital
- Windsor Regional Hospital

**Thank you for taking the time to complete this survey! Your thoughts and feedback are essential for working together to improve the trauma patient’s journey.**
